# Anthropogenic Impacts on Meiosis in Plants

**DOI:** 10.3389/fpls.2018.01429

**Published:** 2018-09-28

**Authors:** Lorenz K. Fuchs, Glyn Jenkins, Dylan W. Phillips

**Affiliations:** Institute of Biological, Environmental and Rural Sciences, Aberystwyth University, Aberystwyth, United Kingdom

**Keywords:** anthropogenic, meiosis, recombination, plants, pollution, evolution

## Abstract

As the human population grows and continues to encroach on the natural environment, organisms that form part of such ecosystems are becoming increasingly exposed to exogenous anthropogenic factors capable of changing their meiotic landscape. Meiotic recombination generates much of the genetic variation in sexually reproducing species and is known to be a highly conserved pathway. Environmental stresses, such as variations in temperature, have long been known to change the pattern of recombination in both model and crop plants, but there are other factors capable of causing genome damage, infertility and meiotic abnormalities. Our agrarian expansion and our increasing usage of agrochemicals unintentionally affect plants via groundwater contamination or spray drift; our industrial developments release heavy metals into the environment; pathogens are spread by climate change and a globally mobile population; imperfect waste treatment plants are unable to remove chemical and pharmaceutical residues from sewage leading to the release of xenobiotics, all with potentially deleterious meiotic effects. In this review, we discuss the major classes of exogenous anthropogenic factors known to affect meiosis in plants, namely environmental stresses, agricultural inputs, heavy metals, pharmaceuticals and pathogens. The possible evolutionary fate of plants thrust into their new anthropogenically imposed environments are also considered.

## Introduction

Humanity’s impact on natural ecosystems is well documented and has led to a decline in biodiversity globally (Cardinale et al., 2012; Hautier et al., 2015). The anthropogenic drivers responsible for this are predominantly related to climate change and pollution that stem from the agricultural and industrial demand to support an ever-growing population. These factors are also known to effect cellular processes, meiosis being particularly vulnerable. The meiotic process is key for all sexually reproducing organisms as it is responsible for halving the chromosome number during gametogenesis and for the process of recombination which generates much of the genetic diversity. The biochemical processes underpinning meiosis are highly conserved but can be influenced by both abiotic and biotic stresses (Modliszewski and Copenhaver, 2017).

Atmospheric and terrestrial pollutants, and xenobiotic compounds (defined as any non-biological compounds that have a detrimental effect on the organism) are prevalent in the environment. The actual number of such compounds is difficult to ascertain accurately, but the United States Environmental Protection Agency currently lists over 85,000 substances on their Toxic Substances Control Act Chemical Substance Inventory (Donner et al., 2010; European Psychiatric Association [EPA], 2018^1^). The genotoxicity of such compounds on plants has been assayed using a variety of methods and a range of plants species including *Tradescantia paludosa*, *Allium cepa*, and *Vicia faba* (Kristen, 1997). The assays involve exposing plants to a known pollutant and assaying the number of chromosome aberrations induced. Initially, assays utilized root cells undergoing mitosis but it was later realized that meiotic cells were far more sensitive to such compounds (Kristen, 1997). The most widely used assay is the *Tradescantia* Micronucleus-in-Tetrad Assay for Environmental Mutagenesis, commonly referred to as the Trad-MCN assay (Rodrigues et al., 1997). The Trad-MCN assay detects micronuclei formed during meiosis, and has been used for *in situ* and *in vivo* laboratory tests to determine the genotoxicity of pollutants in the air, water and soil. The sensitivity of this assay is staggering; for example, plants exposed to various brands of air fresheners for between to 1–6 h results in a significantly higher number of micronuclei (Ma and Harris, 1987a,b). Such assays emphasize the sensitivity of meiosis to external factors.

In some instances, it is desirable to alter the meiotic process, and there has been a resurgence in interest to modulate the recombination landscape in crop plants in order to increase genetic variability for selection in advanced breeding programs. The benefit to plant breeding is clear, but for non-crop plants such destabilizing factors could be detrimental and could confer selective disadvantage. The aim of this review is to examine anthropogenic factors such as climate, agrochemicals, heavy metals, combustible gasses, pharmaceuticals and pathogens, which are known to influence meiosis in a range of plant species (summarized in **Table 1**).

**Table 1:** Summary of anthropogenic factors and their influence on meiosis.

Factor	Species	Phenotype^∗^	Reference
Temperature	*Arabidopsis thaliana*	↑CO	Francis et al., 2007
	*Hordeum vulgare*	↑CO	Phillips et al., 2015
	*Endymion non-scriptus*	↓CO	Elliott, 1955
		↓CO	Wilson, 1959b
	*Hyacinthus orientalis*	↑↓CO	Elliott, 1955
	*Oryza sativa*	↑CO	Si et al., 2015
	*Populus pseudo-simonii*	St, L, M, PM, Sp	Wang et al., 2017
	*Rhoeo spathacea*	↓CO	Lin, 1982
	*Rosa* spp.	Sp	Pecrix et al., 2011
	*Tradescantia bracteata*	↑CO	Dowrick, 1957
	*Triticum aestivum*	PM, L, M	Omidi et al., 2014
	*Uvularia perfoliata*	↑CO	Dowrick, 1957
Water stress	*Zea Mays*	↑CO	Verde, 2003
	*Oryza sativa*	U, L, M	Namuco and O’Toole, 1986
	*Sesbania cannabina*	PM, U, L	Srivastava and Kumar, 2016
	*Hemerocallis*	M	Karihaloo, 1986
		M	Karihaloo, 1994
Salinity	*Arabidopsis thaliana*	↑CO	Boyko et al., 2006
		↑CO	van Tol et al., 2018
Nutrient solution	*Gilia millefoliata* ×*G. achilleaefolia*	↑CO, ↓B	Grant, 1953
	*Solanum lycopersicum*	↑CO	Griffing and Langridge, 1963
	*Triticum aestivum (-Ph1)*	↑CO	Martín et al., 2017
Potassium	*Lolium temulentum*	↑CO	Law, 1963
	*Pennisetum glaucum*	↑CO	Dhesi, 1975
Phosphate	*Secale cereale*	↑CO	Bennett and Rees, 1970
	*Hordeum vulgare*	↑CO	Fedak, 1973
	*Pennisetum glaucum*	↑CO	Dhesi, 1975
	*Festuca pratensis* (2x and 4x)	↓M	Deniz and Tufan, 1998
	*Arabidopsis thaliana*	↑↓CO	Barth et al., 2000
Nitrogen	*Secale cereale* (4x)	↑Bivalent	Hossain, 1978
Herbicide	*Hordeum vulgare*	St, B, M	Wuu and Grant, 1966
		St, B, F, M	Wuu, 1967
		↓CO	Sharma et al., 1981
	*Sorghum vulgare*	A, Sp	Liang et al., 1969
		A, Sp, U	Lee et al., 1974
		A, B, L	Soriano, 1984
		Multiple nucleoli, PM	Currie and Liang, 1996
	*Vicia faba*	St, L, B, M, A	Amer and Ali, 1974
		St	Badr et al., 1987
	*Triticum durum*	B, L	Razu et al., 2014
Insecticide	*Hordeum vulgare*	St, B, F, M	Wuu, 1967
	*Vicia faba*	St, B, L	Amer and Ali, 1968
		St, B, L	Amer and Farah, 1968
		St, B, L, Sp	Amer and Farah, 1976
		St, B, F, L, U, Sp, M	Amer and Farah, 1980
		St, L, B	Amer and Farah, 1983
		St, B, F	Amer and Farah, 1987
	*Capsicum annuum*	St, B, L, Sp, M	Devadas et al., 1986
		St, B, L, M, U	Reddy and Rao, 1981
		↓CO, S, B, L, U, M	Lakshmi et al., 1988
	*Allium cepa*	St, B, L, PM	Kuchy et al., 2016
Fungicide	*Hordeum vulgare*	St, B, F, M	Wuu, 1967
		↓CO	Bennett, 1971
		↓CO	Sharma et al., 1983
	*Allium cepa*	St, B, F, M	Mann, 1977
		St, B, L, M	Fisun and Rasgele, 2009
		St, B, L, PM	Kuchy et al., 2016
	*Pisum sativum*	↑↓CO	Choudhary and Sajid, 1986
	*Capsicum annuum*	St, B, L, U, multivalents	Prakash et al., 1988
	*Lathyrus sativus*	S, B, L, F, U	Kumar and Sinha, 1991
Heavy Metals	*Glycine max*	St, L, U, B, PM	Kumar, 2007
	*Zea mays*	PM, B, L, U, F, M	Kumar and Rai, 2010
	*Vicia faba*	St, L, B	George, 2000
	*Hordeum vulgare*	PM, L, B, M	Mittal and Srivastava, 2014
	*Lathyrus sativus*	U, ↓CO	Kumar and Ritambhara, 2006
		St, Sp, L	Tripathi and Girjesh, 2010
	*Capsicum annuum*	St, PM, L, B, F, M	Aslam et al., 2017
	*Vicia faba*	S, St, L, B	George, 2000
Caffeine	*Secale cereale*	L, B, ↓CO	de la Peña et al., 1981
	*Trigonella foenum-graecum*	L, B, U, St, PM	Anis and Wani, 1997
Pathogen	*Oryza sativa*	↑CO	Si et al., 2015
	*Carica papaya*	L, B, U	Pandey, 2017
	*Datura quercifolia*	↓CO, U, L, M	Kaul, 1968
	*Solanum lycopersicum*	PM, A	Caldwell, 1952
	*Capsicum annuum*	↓CO, Sp	Swaminathan et al., 1959
	*Lycopersicon esculentum*	B, PM, M, F	Andronic, 2012
	*Hordeum vulgare*	B, PM, L	Andronic, 2012
Antibiotics	*Allium cepa*	St, L, P, B, F	Mann, 1978
	*Lathyrus sativus*	U, St, B	Kumar and Sinha, 1991


*^∗^CO, change in crossover rate; St, stickiness; L, laggards; B, bridges; F, fragments; U, univalents; A, aneuploidy; M, micronuclei; PM, precocious movements; Sp, spindle aberrations. ↑ denotes increase, ↓ denotes decrease.*

## Environmental Stresses

It is now generally accepted that human activity is responsible for climate change (International Plant Protection Convention [IPPC], 2014), which is manifested as a rise in global temperature and carbon dioxide levels, more extreme and unpredictable weather patterns, and a rise in sea levels with its concomitant increased risk of salinisation of ground water. These changes are very likely to challenge our agricultural productivity and threaten global food security. As a consequence, it is imperative that we understand the plant’s response to abiotic stresses, as this will inform our strategies of intervention to protect and adapt future crops (Mickelbart et al., 2015). This section focusses upon the effects of environmental stresses on meiosis and recombination in some model, crop and non-crop species. It is pertinent to consider this process in this context, since recombination is fundamental to the fertility, genetic stability and genetic potential of sexually reproducing organisms.

Through exhaustive investigation, we now have a very good idea of how meiosis works (Wang and Copenhaver, 2018), and how external stresses may invoke certain adaptive and ameliorative responses in plants (De Storme and Geelen, 2014; Bomblies et al., 2015; Modliszewski and Copenhaver, 2017). Suboptimal high and low growth temperatures and their effects on modulating crossover (CO) frequency and distribution in plants have received particular attention, and have long been recognized in plants. In most instances, such adjustments in CO landscape have been inferred from observing chiasmata, the cytological equivalents of COs at metaphase I. Elliott (1955) showed that there was a reduction of CO frequency in meiosis in *Endymion nonscriptus* at 20°C but not between 1 and 15°C. These observations are not consistent with a subsequent study, which showed a consistent but gradual decrease in mean chiasma frequency with increasing temperature in this species (Wilson, 1959b). This difference could be explained by genetic and environmental influences beyond temperature. Dowrick (1957) recorded an increase in interstitial chiasmata with increasing temperature in *T. bracteata* and *Uvularia perfoliata*, Elliott (1955) showed a detrimental effect on chiasma frequency at 20°C in *Hyacinthus orientalis*, and Lin (1982) showed that chiasma frequency is reduced at 37°C in *Rhoeo spathacea*. A combination of water and temperature stress in two varieties of *Hemerocallis* induces desynapsis (Karihaloo, 1986, 1994). High temperature induces meiotic irregularities and diploid pollen formation in species of *Rosa* (Pecrix et al., 2011) and *Populus* (Wang et al., 2017; Tian et al., 2018), which is considered to have important implications in terms of adaptation and evolution through polyploidisation. Recombination frequencies in *Arabidopsis thaliana* are positively correlated with temperature over the range 19–28°C (Francis et al., 2007), but this response appears to be part of a U-shaped curve in which chiasma frequencies rise from a low at 18°C to higher values at both 8 and 28°C (Lloyd et al., 2018). The changes involve class I interfering COs only (Modliszewski et al., 2018), in contrast to the observations in barley (*Hordeum vulgare*) described below.

The studies above describe temperature effects on recombination in non-crop species. Whilst these have value in forwarding our knowledge and understanding of fundamental biological processes, they cannot substitute for studying these effects in the crops themselves, especially given the variation in responses of different plants, which confounds direct translation from one species to another. Unfortunately, there is a dearth of systematic studies of this phenomenon in crop plants, but several particular crops stand out. Prakken (1943) showed that high temperature and drought together exacerbated reduced bivalent formation in asynaptic rye, and Saini et al. (1984) showed that temperature and water stress together caused male sterility in wheat, but not through any demonstrable negative effect on meiosis. The latter contrasts with more recent observations, which show that heat stress induces meiotic chromosomal abnormalities in four wheat cultivars, and various changes in meiotic defects in some cereal crops (Rezaei et al., 2010; Omidi et al., 2014). Si et al. (2015) showed that some but not all rice (*Oryza sativa*) plants subjected to heat stress had higher recombination frequencies. Powell and Nilan (1963) described by cytology significantly higher chiasma frequencies at higher temperatures in an inversion heterozygote of barley. In contrast, Jensen (1981) used genetic mapping of barley to show that temperatures of 12, 18, and 24°C had no effect on recombination frequencies. Higgins et al. (2012) later reported that a rise in temperature not only increases the CO frequency, but also redistributes COs to more distal chromosomal locations. Since COs are highly distally localized in this species, this phenomenon has important implications for cracking open tight linkage groups, which are otherwise refractory to recombination events. These observations were confirmed by a subsequent study (Phillips et al., 2015), which went on to show that high temperature increases only class II CO frequency in male meiosis, and also demonstrated that interstitial regions of the genome are more prone to these changes. There is a tantalizing prospect, therefore, that simple heat shocking of barley at vulnerable stages of development could release potentially useful genetic variation for use in advanced breeding programs. However, this is predicated upon a greater understanding of the genetic (see review by Wang and Copenhaver, 2018) and epigenetic processes (reviewed by Yelina et al., 2015) underpinning these effects, which could ultimately enable the precise reprogramming of the crop.

The variation in response to temperature, even within the same species, may indicate that there is plasticity in the mechanisms governing CO control, or may implicate alternative pathways with different mechanisms. A caveat is that these differences may simply reflect discrepancies in the methods used to acquire and compare data, as has been inferred by (Wilson, 1959a; Bomblies et al., 2015; Lloyd et al., 2018).

Whilst temperature effects on recombination are the most widely described in the literature, there is some information describing other abiotic factors of relevance to climate change. Water stress has been shown to cause meiotic chromosome abnormalities in rice, such as laggards, micronuclei, univalents and a partial arrest of meiosis at severe stress levels (Namuco and O’Toole, 1986). In water stressed barley, abnormal chromosomal pairing and segregation during meiosis was found, leading to loss of pollen fertility (Skazkin and Zavadskaya, 1957). Cytological studies in *Sesbania* pea found that waterlogging stress resulted in various chromosomal aberrations and a reduction in pollen fertility (Srivastava and Kumar, 2016). Verde (2003) has also presented evidence that meiotic recombination increases in response to droughting in two genotypes of maize (*Zea mays*). van Tol et al. (2018) detected a 70% increase in recombination frequency between markers in response to salt stress of *Arabidopsis*, and genotyping revealing that CO fluctuation was not limited to the region between the marker genes but occurred throughout the genome. Modliszewski et al. (2018) did not detect the same effect under similar conditions in the same species. Considering that elevated CO_2_ levels is one of the most prominent causes of climate change, it is surprising that little research has been conducted to examine its influence on meiosis. Koti et al. (2005) showed no effect of elevated CO_2_ on pollen viability in *Glycine max*, inferring that meiosis was also unaffected.

## Agricultural Inputs

Innovations that emerged during the ‘Green Revolution’ led to a steady rise in agricultural output across the globe (Tilman et al., 2002). These gains were driven principally by the development of new crop varieties and through the increased use of inputs, namely synthetic fertilizer and pesticides. The benefits of these inputs to crop productivity are clear and well documented, as are the negative impacts on the adjoining environment. Agricultural pollutants can influence natural non-target plant populations in close proximity via direct contact (e.g., spray drift), or may affect a much larger area via groundwater contamination (Moss, 2008).

## Fertilizer

The global demand for fertilizer nutrients (N, P_2_O_5_, and K_2_O) has increased steadily since the 1960s, and is predicted to increase from 184.02 million tons in 2015 to over 200 million tons in 2020 (Food and Agriculture Organization [FAO], 2017). It has long been recognized that nutrient state influences meiotic processes. One of the first studies used a sterile F_1_ hybrid between *Gilia millefoliata* and *G. achilleaefolia*, and observed that plants grown in rich loam had consistently higher bivalent frequencies, more chiasmata per bivalent on average, and fewer anaphase bridges than those grown in sand (Grant, 1953). Later, Griffing and Langridge (1963) determined the optimal growth conditions for elevated levels of recombination in tomato, a key component in commercial breeding programs. They observed that the CO frequency for a known interval decreased from 17 to 6% over a 6 months period during which no additional fertilizer was supplied. Subsequent addition of fertilizer restored the CO frequency to 12%, implying that nutrient-rich conditions enhance the rate of CO (Griffing and Langridge, 1963).

Subsequent investigations used a more systematic approach in order to isolate particular components, which had the greatest influence on meiosis. In an early example of such an approach, Law (1963) determined the influence of high and low concentrations of potassium and calcium on chiasma frequency in *Lolium temulentum* grown at both 20 and 30°C. High potassium increased chiasma frequency at both temperatures, and reduced its variance in the high temperature regime (Law, 1963). Bennett and Rees (1970) observed subsequently the same phenomena in rye (*Secale cereale*) grown under high phosphate conditions, recording higher chiasma frequencies and lower variance compared with controls. One of the most detailed studies of the effects of phosphate was conducted in *Arabidopsis* by comparing the recombination rate between pairs of genes conferring resistance to kanamycin and hygromycin (Barth et al., 2000). Under 10-fold higher levels of phosphate, recombination between loci on chromosomes 1 and 2 was significantly reduced, whilst intervals on chromosomes 4 and 5 showed minor, non-significant increases in recombination.

High phosphate also has a notable effect on a desynaptic barley mutant, enhancing the formation of chiasmata to 10.6 per cell compared to 7.7 for the control (Fedak, 1973). In a subsequent study, Dhesi (1975) noted a significant increase in chiasmata at metaphase I in a desynaptic mutant of pearl millet (*Pennisetum glaucum*) subjected to elevated levels of phosphate or potassium. However, elevated phosphate does not influence recombination in all species, such as soybean (*G. max*) (Hanson, 1961).

All of the studies described above used diploid plant species. However, Grant (1953) reported that bivalent frequency dramatically increases in a neoallotetraploid formed from the hybridisation between *G. millefoliata* and *G. achilleaefolia* when watered with a solution of mineral nutrients. Autotetraploid rye grown in Hoagland’s solution II containing nitrogen has a higher quadrivalent frequency, at the expense of bivalent formation, and the same number of chiasmata compared to plants grown without nitrogen (Hossain, 1978). More recently, Hoagland’s solution has also been shown the significantly increase CO formation in wheat and wheat-rye hybrids lacking the *Ph1* locus, which suppresses COs between homoeologs (Martín et al., 2017). A subsequent study showed that the magnesium in the Hoagland’s solution was responsible for the observed phenotype (Rey et al., 2018).

## Pesticides

Crop protection agrochemicals are another cornerstone of modern agriculture and are essential for minimizing yield losses. In 2015 alone, a total of 2,752,759 tons of active product was applied to crops globally, predominantly as herbicides, fungicides, and insecticides (Food and Agriculture Organization [FAO], 2018^2^). The influence of each of these pesticides groups on meiosis has been assessed (Sharma and Panneerselvam, 1990) and key examples are highlighted below.

One of the earliest studies to examine the influence of herbicides on meiosis was conducted by Wuu and Grant (1966). Barley seeds were soaked in 3-(3,4-dichlorophenyl)-1-methoxy-1-methylurea for 24 h prior to sowing, and meiocytes collected from the treated plants were analyzed cytologically. The authors documented numerous defects such as chromatin bridges, micronuclei, and asynchronous division. Sharma et al. (1981) examined the influence of three additional herbicides, bromacil, lenacil, and terbacil, all applied to barley seed at various concentrations for 6 h. Treatment with terbacil significantly reduced the chiasma frequency and was as disruptive as ethyl methanesulfonate (EMS), which was included as a control compound. The susceptibility of meiosis in *V. faba* to 2, 4, 5-trichlorophenoxyacetic acid (2,4, 5-T), 2, 4-dichlorophenoxyacetic acid (2, 4-D) and 2, 4-dichlorophenol, an intermediate product in the degradation pathway of 2, 4-D, was assessed by Amer and Ali (1974). The compounds were applied to both seed and sprayed onto 15 and 35 days old plants. Treatment at 35 days with 2, 4, 5-T led to the highest levels of sterile pollen grains resulting from stickiness, lagging chromosomes, and chromosome fragmentation during meiosis. Badr et al. (1987) reported terbutryn also induced chromosomal abnormalities in 11.3% of cells undergoing meiosis in *V. faba*.

Liang et al. (1969) sprayed sorghum (*Sorghum vulgare*) plants that ranged between 5 and 20 cm in height with atrazine (2-chloro-4-ethylamino-6-isopropylamino-s-triazine), 2,4-D, alkanolamine salts of 2,4-D, and non-phytotoxic petroleum oil (crop oil), or their combinations. All increased the level of cytological aberrations at meiosis, including inducing aneuploidy and polyploidy. Two further studies assessed the influence of atrazine on meiosis in sorghum. Lee et al. (1974) also found a high degree of meiotic abnormalities in atrazine-treated sorghum, while a subsequent study noted more subtle changes to the meiotic nuclear landscape; additional nucleoli were frequently observed at diplotene and early diakinesis in treated plants (Currie and Liang, 1996). Soriano (1984) also reported the influence of a herbicide, whose active ingredient is N-(butoxymethyl)-2-chloro-2′, 6′-diethyl-acetanilide, applied at concentrations of 0.05–0.20% to sorghum seed. The lowest concentration induces aneuploidy in 2.9% of metaphase I cells, increasing to 8.8% at the highest concentration, compared to 0% in untreated plants. Interchanges, bridges and fragmentation were observed during meiosis I only at the higher concentrations.

The herbicide maleic hydrazine was applied to seed of *Helianthus annuus* at concentrations of 10^-5^ M to 10^-2^ M to determine its meiotic influence (Kaymak, 2005). This study reports not only a significant increase in abnormalities during both the first and second meiotic divisions, even at the lowest concentration tested, but also identifies a significant degree of abnormalities in the subsequent generation. A more recent study by Singh and Srivastava (2014) tested the effect of glyphosate and pendimethalin applied to the foliage of *Vigna mungo* 21 days from the point of sowing. They noted numerous defects, including chromatin bridges, laggards at anaphase I, disturbed spindle formation and binucleate cells in 17% and 21% of pollen mother cells (PMCs) treated with pendimethalin and glyphosate, respectively.

The influence on meiosis of a wide range of insecticides has been assayed in *V. faba* (Amer and Farah, 1968, 1976, 1980, 1983; Amer and Ali, 1983). In one of the earliest studies, Amer and Farah (1968) applied *N*-methyl-1-naphthyl-carbamate over various timeframes. After a single application, 8.7% of PMCs contained an aberration at diakinesis and metaphase I, compared to 1% in the control, rising to 23% if applied daily over a 7-day period. Defects were also later observed at anaphase I, metaphase II, and anaphase II (Amer and Farah, 1968). In *V. faba* both *O*,*O*-dimethyl-*N*-methyl-carbamidomethyl dithiophosphate and *O*-isopropyl-*N*-phenyl carbamate applied to seed prior to planting or sprayed (on day 15 or 35) induced multipolar anaphase II, along with a host of other types of meiotic abnormalities, the effects of which did not influence the yield phenotype of the successive two generations (Amer and Farah, 1976). One of the most profound effects was noted for the organophosphate insecticide chlorpyrifos (0,0-diethyl 0-3, 5, 6-trichloro-2-pyridyl phosphorothioate). When applied as a spray to seedlings or at the flowering stage the result was the same; chromosome stickiness was observed in more than 80% of the abnormal meiocytes (Amer and Farah, 1983). A single application during flowering of another organophosphate, methamidophos (0,*S*-dimethyl phosphoramidothioate), was sufficient to significantly increase the number of abnormal PMCs to 8.4%, compared to 1% in the control (Amer and Farah, 1987). Repeat application exacerbates the effect, causing abnormalities in 21% of PMCs.

The meiotic influence of four organophosphorus insecticides applied to *Capsicum annuum* was assessed by Devadas et al. (1986). The insecticides, namely dimethoate, DDVP, phosphomidon and monocrotophos were applied to seed at concentrations of 0.1, 0.5, and 1.0% for 24 h. Each treatment depressed pollen viability, with the degree of sterility rising with increasing concentration. The sterility is caused by univalents, multivalents, bridges, lagging chromosomes, non-synchronization, multipolar formation, micronuclei, unoriented and unequal disjunction of chromosomes identified in the preceding meiosis. The authors also claim that the insecticides tested are as disruptive as ionizing radiation. Reddy and Rao (1981), also working with *C. annuum*, focused on two different compounds, BHC and Nuvacron, the latter being a organophosphorus systemic and contact insecticide. The compounds were applied at a range of concentrations to both the seed and sprayed on plants at fortnightly intervals. Both were found to induce abnormalities during meiosis, ranging from 3.1 to 5.6% for BHC and 7.7–15.6% for Nuvacron. The authors note that the lowest concentration is favored by Indian farmers at the time of publication. A later study by Lakshmi et al. (1988) examined the influence of Ekalux EC 25 [0-diethyl-o-(quinoxylinyl-2) phosphorothionate] and Metasystox (oxydemeton-methyl = S-(2(ethyl-sulphinyl)-ethyl) & 0, 0-dimethyl phosphorothioate), applied to seed of *C. annuum* followed by four spray applications during growth. This study is one of few to score chiasma frequency, and noted a steady decline in COs with increasing concentration of each insecticide. COs were reduced to the lowest number of 21.08 for Ekalux EC 25 and 20.5 for Metasystox, compared to 23 in the control. Other meiotic defects including laggards, bridges, stickiness and univalents were also noted in this study.

Kuchy et al. (2016) examined the effects on *A. cepa* of an organophosphate insecticide containing dichlorvos (2,2-dichlorovinyl dimethyl phosphate) and a organochlorine insecticide, endosulfan. The organophosphate compound was the most potent at inducing abnormalities, affecting 11–27% of PMCs depending upon which concentration was applied, compared to 3% in the control. Endosulfan also caused numerous meiotic failures, with 9–20% PMCs containing defects, depending upon the concentration used. The most common abnormality observed was stickiness at metaphase I and II.

One of the earliest investigations of the influence of fungicides on meiotic recombination was conducted by Bennett (1971) who tested Ethirimal (5-butyl-2-ethylamino-4-hydroxy-6-methyl pyrimidine), a systemic compound applied as a seed dressing in barley. The mean chiasma frequency in each of three cultivars was significantly reduced to 2–4% lower than the control. A later study in the same species showed that the systemic fungicides fuberidazole, carboxin, and oxycarboxin (but not thiabendazole) all significantly reduced chiasma frequency (Sharma et al., 1983). Carboxin and oxycarboxin has an effect even at the lowest concentration tested.

The transgenerational effect of the fungicide carbendazim (2-methoxy-carbamoyl benzimidazole), was studied in two different cultivars of *Pisum sativum* (Choudhary and Sajid, 1986). Plants were sprayed four times during development with the recommended dose of 0.2% aqueous solution, and at 0.4%. Both concentrations significantly altered the chiasma frequency, with one cultivar being more susceptible, implying a genotypic interaction with the fungicide. The subsequent two generations of the treated plants, which received no further applications of fungicide, also had a significantly altered CO landscape, one cultivar having fewer COs contrasting with the second which had more. Abnormalities such as stickiness, fragmentation and micronuclei, were also observed in PMCs of *A. cepa* treated with either one of four different Dithane based fungicides or treated with carbendazim (methyl-2-benzimidazole carbamate-MBC) (Mann, 1977; Kuchy et al., 2016). Fisun and Rasgele (2009) treated *A. cepa* bulbs with roots 1.5–2 cm in length with different concentrations (1800–6000 ppm) of the fungicide tebuconazole, for 3–24 h. All treatments resulted in a significant increase in meiotic abnormalities, including a significant increase in the number of quadrivalents, believed to be a result of chromosome translocations.

## Other Anthropogenic Influences

Whilst environmental stresses and agricultural pollutants commonly affect meiosis in plants, there are also a number of other anthropogenic factors that can have a similar effect. As the human population increases and encroaches upon the global natural environment, the trail of anthropogenic influences grows commensurately. Increasing agricultural and industrial developments expose plant populations to new and different chemical and physical agents and places them in environments that can potentially alter their development. There is greater pollution from industrial and automotive combustion, commercially and pharmaceutically used solvents, additives, chemicals and an increase in pathogen prevalence worldwide (Evans et al., 2008; Gaffney and Marley, 2009; Tornero and Hanke, 2016).

## Heavy Metals and Combusted Gasses

Heavy metal contamination of the environment is increasing due to human activity, with many sources of pollution primarily entering water courses and polluting the land. The accumulation of heavy metals and metalloids in soil also originate from other sources, such as emissions from industrial areas, leaded fuels and paints, sewage sludge, biosolids, animal manures, spillage of petrochemicals and combustion residues (Khan et al., 2008; Zhang et al., 2010).

Samples of agricultural soils from the siling reservoir watershed in Zhejiang Province and an area of north east China that has been of intensively farmed for decades were found to contain high levels of cadmium contamination (Naveedullah et al., 2013; Shan et al., 2013). At some metalliferous sites there can be 100 times elevated metal concentrations in the soil compared with uncontaminated areas (Bert et al., 2002). Soil samples from agricultural areas of Jiaxing, a rapidly industrializing area in the Yangtze Delta of China, has localized hot spots of pollution of copper, zinc, lead, chromium, nickel and cadmium (Xu et al., 2014). The concentrations of the heavy metals lead, copper, cadmium, and zinc can be further increased by urban runoff or combustion sources, and copper, chromium and tin levels even further increased by automobile break and tire wear (Davis et al., 2001; Hulskotte et al., 2007; Shan et al., 2013). Another surprising pollution source are shooting ranges as the soil there often has higher levels of antimony, nickel, lead, copper and zinc and can be used for animal grazing when not in use or decommissioned (Robinson et al., 2008; Bannon et al., 2009).

There may be no cause for concern for some current levels of heavy metal contamination of the soil, and potentially the food chain, as they may often be below the acceptable thresholds for safe human consumption. There is, however, reason to investigate what effects they may have on the recombination landscape of the plants and crops that grow in these areas, as heavy metals are known to effect plant development in a number of ways. *Arabidopsis* plants treated with 50 or 100 mM copper, cadmium or nickel have at least a twofold increase in somatic homologous recombination frequency with successive treated generations exhibiting even higher increases (Rahavi et al., 2011). This study also found that the subsequent generation, which was not exposed to any stress, still possessed significantly higher residual rates of recombination when compared to the non-exposed progeny. Further studies found that chromosomal abnormalities, including chromosomal bridges, scattering and precocious movements occurred in *G. max* and *Z. mays* when treated with cadmium (Kumar, 2007; Kumar and Rai, 2010). Similar results were found when *H. vulgare* was treated with a combination of cadmium and chromium. This combination treatment also resulted in a significant decrease in the number of pollen grains per anther and a significant increase in pollen sterility (Mittal and Srivastava, 2014).

Although the current known levels of heavy metals found in soils due to anthropogenic influences may not be enough to modify the recombination landscape alone, there are other agents, such as anthropogenic gasses, that are known to have detrimental effects on plants. A cytogenetic test based upon quantifying micronuclei resulting from chromosome breakage in meiotic pollen mother cells *T. paludosa* (Trad-MCN) has found that various anthropogenic gasses such as NO_2_, SO_2_, O_3_, HN_3_, and EMS have clastogenic effects. Both gaseous HN_3_, and EMS were clastogenic after 6 h exposures while SO_2_ and NO_2_ required 22 and 24 h, respectively (Ma et al., 1982; Rodrigues et al., 1996). Several indoor environments containing pipe and tobacco smoke, and formaldehyde fumes were found by the Trad-MCN assay to cause chromosomal breakage, as well as several outdoor locations such as parking garages, truck and bus stops, agrochemical industrial sites, a P-dichlorobenzene treated herbarium and an industrial site (Ma et al., 1980, 1982; Ma and Harris, 1987a,b). Although this only shows that *T. paludosa* can be clastogenically affected by anthropogenic environments, it emphasizes the unintended consequences of human activities.

Heavy metals and combusted gasses in the environment are not the only anthropogenic sources that could change the recombination landscape of crops around the world; there are also large amounts of pharmaceutical chemicals that pass into the environment and to the soil.

## Pharmaceutical Chemicals

As pharmaceutical drug use increases worldwide, there is growing concern for the high level of pharmaceutical residues in aquatic environments (Uslu et al., 2013). Treatment plants are not always able to remove pharmaceutical residues from sewage completely and often release amounts into the receiving waters (Heberer, 2002; Gaw et al., 2014; Küster and Adler, 2014; Zhang et al., 2016). While a large part of the concern comes from the potential of these residues to affect aquatic life and enter the drinking water supply, there is justification to question the effects they could have on plant life when treated biosolids are applied to agricultural land.

Extremely low levels of the anti-cancer drug bleomycin, known to cause an increase in DSBs and increased somatic recombination, have been found at concentrations of 11 – 19 ng/L and <5–17 ng/L in sewage treatment effluent and rivers, respectively (Aherne et al., 1990). Whilst these doses are well below the normal chemotherapeutic doses administered (20–30 mg/m^-2^), data are sparse concerning the bioaccumulation of bleomycin or the amount that it is used worldwide. Qi et al. (2014) showed that 98 tons of pesticides, 152 tons of pharmaceuticals, 369 tons of polycyclic aromatic hydrocarbons and 273 tons of household and industrial chemicals are flushed annually into the East China Sea by the Yangtze river. This level of pollution could potentially affect meiotic recombination in plants, but virtually no investigations have been undertaken. The recent significant increase in drug-resistant bacterial strains is often attributed to the indiscriminate use of antibiotics by today’s society. However, there should perhaps also be concern about the bioactivity of waste antibiotics in the environment. Some antibiotics in water courses and agricultural land are known to affect plants. For example, ciprofloxacin has been found in municipal waste water (Lee et al., 2007) and in soil samples (Golet et al., 2002; Goulas et al., 2016) and is known to cause double strand DNA breaks in *Arabidopsis*, (Rowan et al., 2010). Ciprofloxacin’s bactericidal action comes from the inhibition of topoisomerase II (DNA gyrase) and topoisomerase IV, required for various bacterial DNA processes including replication, transcription, repair, strand supercoiling repair, and recombination (Aldred et al., 2014), and a recent study found that ciprofloxacin targets *A. thaliana* gyrase (Evans-Roberts et al., 2016). Tetracycline has been shown to induce meiotic aberrations including stickiness, laggards, bridges, and fragments in *A. cepa* (Mann, 1978), and has been found in soil fertilized with liquid manure and in soil and water near intensive commercial livestock operations (Hamscher et al., 2002; Thiele-Bruhn, 2003).

The increase in anthropogenic chemicals in the environment is not limited to pharmaceuticals, but also applies to chemicals resulting from human consumption. Caffeine can be found in soil due to the reuse of treated wastewater for irrigation (Bruton et al., 2010; Williams and McLain, 2012). Anis and Wani (1997) found that caffeine-treated populations of *Trigonella foenum-graecum* exhibited several meiotic abnormalities including laggards, univalents, bridges, stickiness, and precocious chromosome movements. A 0.1% caffeine solution was administered to *S. cereale* and abnormalities such as laggards, bridges and fragments and decreased chiasma frequency were observed (de la Peña et al., 1981). The widely used chemical bisphenol A (BPA) affects microtubule arrays of meristematic root-tip cells of *P. sativum* resulting in the stalling of cytokinesis, deranged interphase and mitotic microtubule arrays, and abnormal chromosome segregation (Adamakis et al., 2013).

## Pathogens

The full impact climate change will have on the prevalence and spread of virus disease epidemics in natural vegetation and cultivated plants and crops is still unknown. Research suggests that elevated environmental CO_2_ levels can alter hormone production in plants and precipitate the observed shift in susceptibility to insect herbivores and pathogens (Casteel et al., 2012; Zhang et al., 2015). Whilst research to understand the possible effects of some common climate change scenarios is ongoing, reviewed extensively in Jones (2016) and Trebicki et al. (2017), we still do not know what impact climate change may have on the recombination landscape of plants. Climatic changes are compounding the threat of spread of plant pest and diseases. A recent analysis indicated that crop pests are moving 2.7 km poleward annually and that on average, 10% of the major plant pests and disease agents have already infested half of the countries that they potentially could infect (Bebber et al., 2013, 2014). *Carica papaya* infected with papaya ring spot virus exhibited an increase in laggard chromosomes, and had a lower mitotic index and worse pollen viability compared to its healthy counterpart (Kumar Ravindra, 2017). Infection of *Arabidopsis* with the oomycete pathogen *Peronospora parasitica* has been shown to lead to an increase in somatic recombination frequency (Lucht et al., 2002). Interestingly, when Molinier et al. (2006) treated *Arabidopsis* with flagellin, an elicitor of plant defenses, they found that not only did somatic homologous recombination increase in the treated individual but also in successive generations. *Nicotiana tabacum* treated with the tobacco mosaic virus behaves in a similar way; somatic recombination increased in both the subject and its offspring, with one study showing that the offspring even exhibited a delay in symptom development when infected with viruses (Kovalchuk et al., 2003; Kathiria et al., 2010). Si et al. (2015) found that in some of the F_2_ generation of *O. sativa* treated with rice blasts spores there was a significantly higher number of CO events compared to the controls. *Datura quercifolia* treated with mosaic virus resulted in a drastic decrease in chiasma frequency, and the presence of univalents led to many irregularities such as laggards, micronuclei and a significant reduction in pollen and seed fertility (Kaul, 1968). Caldwell (1952) noted that *Solanum lycopersicum* treated with mosaic virus led to the breakdown of meiosis and in some instances cells forming with an irregular number of chromosomes. Yao et al. (2013) found that a local infection of either tobacco mosaic virus or oilseed rape mosaic virus leads to a systematic increase in somatic homologous recombination frequency, with older plants having a higher recombination frequency than younger plants. *Lycopersicon esculentum* and *H. vulgare* infected with barley stripe mosaic virus exhibited an increase in various chromosome abnormalities and a shift in chiasmata toward the interstitial regions (Andronic, 2012). Anthropogenic influences resulting in a changing climate can lead to an increased level of viral vectors and more disease epidemics in plants worldwide, but they could also be enough to change the meiotic recombination landscape forever.

## Limitations of Current Studies

As outlined above, there is a wide range of anthropogenic factors that have been shown to cause various meiotic abnormalities. In the vast majority of cases, the precise biochemical action induced by the treatment during meiosis has not been ascertained; temperature is the notable exception (see earlier section for detail). Many studies reported abnormal meiocytes with chromosome stickiness, bridges, fragmentation and micronuclei. However, it is yet to be determined if the factors themselves are clastogenic and cause DNA damage directly, or whether they interfere with the repair of double strand breaks (DSBs) formed naturally during prophase I. Cytology of fixed meiotic material was by far the most prevalent method of determining the extent of meiotic abnormalities induced by each treatment, and in most studies only gross changes were recorded. More subtle influences, such as changes in chiasma frequency at metaphase I, were recorded in only approximately a third of the studies summarized in **Table 1**. The scoring of chiasmata is not sensitive and cannot detect small changes in recombination frequency, making it likely that more subtle effects at the lower concentration range were not detectable.

The biochemical pathways affected by these treatments may be elucidated by studies in non-plant species. Allard and Colaiacovo (2010) used *Caenorhabditis elegans* to analyze the meiotic molecular pathways affected by BPA. They showed that BPA perturbs both synaptonemal complex and chromosome integrity during pachytene, and also alters DSB repair progression and activation of the DNA damage checkpoint. The effect of the herbicide atrazine on meiosis has been studied in both male and female mice (Gely-Pernot et al., 2015, 2017). In male mice, atrazine reduced sperm count by 68%, caused by a delay in meiotic progression arising from the persistence of unrepaired DSBs (Gely-Pernot et al., 2015). The study also found that the herbicide affects the expression of genes involved in mitochondrial function, steroid-hormone function and GTPase activity, and also altered the pattern of histone H3 trimethylation at lysine 4 (H3K4me3). A subsequent study switched focus to female meiosis and found that atrazine increased the level of oxidative stress in the nuclei of meiotic cells, as measured by the level of 8-oxo-guanine, which affected DBS repair, synapsis and CO frequency (Gely-Pernot et al., 2017).

Another shortcoming of most published studies is that the concentrations of the chemical treatments used cannot be related to those used in agriculture or experienced by plants in natural environments. The biological significance, in terms of potential threat to the meiotic process, is therefore difficult to ascertain. Taking agricultural inputs as an example, a number of agrochemicals have been subsequently banned or their application drastically reduced. The persistence of these substances in the environment also varies, and may have the potential to affect plant communities long after their last application. For example, atrazine was first introduced as a herbicide in 1958 but was subsequently withdrawn from use in most Northern European countries in the early 1990s, and banned from the whole European Union in 2004. Traces of atrazine are still detectable in soil sampled from agricultural land in Germany where the last application was prior to 1991, and in marine sediments of the Mediterranean (Noedler et al., 2013; Vonberg et al., 2014).

In the majority of studies cited, only one treatment was applied to a single plant species that was grown under controlled conditions. This is starkly different to reality where multiple xenobiotics, abiotic and biotic stresses may impact together in one environment. To date, only one published study examines the impact on meiosis of plant species growing in polluted environments. Zohair et al. (2012) sampled 14 species belonging to the Cyperaceae and Poaceae growing in the vicinity of industrial sites and agricultural fields around Karachi, Pakistan, and compared them with their counterparts in unpolluted environments. All of the plant species collected from the contaminated sites had significantly higher numbers of aberrant PMCs with precocious movement, stickiness, aberrant spindles or lagging chromosomes. The prevalence of abnormal PMCs varied greatly between species; the largest effect was observed in *Cyperus arenarius* where 99% of PMCs contained defects, compared to 13% for the control, contrasting with *Ochtochloa compressa* where only 7% of PMCs were defective, compared to 2% in the control. The study also identified an elevated number of unreduced dyads and sterile pollen grains.

Numerous genetic modifiers of recombination have been identified. One of the first studies to note this was Gale and Rees (1970) who observed small but significant differences in chiasma frequencies in five *Hordeum* species, attributed to genotypic variation in the populations. Ziolkowski et al. (2017) identified that 56.9% of CO variation in a F_2_ population from a cross between two *Arabidopsis* accessions, Columbia and Landsberg, was caused by semi-dominant polymorphisms in *HEI10*, a conserved ubiquitin E3 ligase. Interaction between such genetic elements and environmental variables has not been extensively studied. Rezaei et al. (2010) noted an interaction between the extent of meiotic irregularities and environmental conditions in *Tritium turgidum*. Zheng et al. (2014) reported that cyclin-dependent kinase G1 (*CDKG1*) was required for normal levels of synapsis and CO formation in male meiosis in *Arabidopsis*, but only at elevated temperatures.

The range of plant species assessed for their meiotic sensitivity to anthropogenic factors is very narrow and confined to angiosperms, and in most instances only those used in agriculture. Single species are usually examined as targets for agrochemicals, and most non-target species are ignored. The effects on angiosperms in non-agricultural habitats, such as woodlands or estuaries and other plant groups, such as gymnosperms, are less known because of the difficulties of experimenting with such species. One such study by Bykova et al. (2018) found that a 27% reduction in precipitation resulted in a 35% reduction in viable pollen grains compared to the control in male *Quercus ilex*. Most of the studies relate to male meiosis, so the sensitivity of the ovule is largely unknown. Hundreds of xenobiotic compounds have been assessed, including those tested by the *Tradescantia* Trad-MCN assay, but this represents a small fraction of the 10,000s of compounds present in the environment (Donner et al., 2010; European Psychiatric Association [EPA], 2018).

## Evolutionary Consequences

Empirical research has shown that both the number and distribution of recombination events are tightly controlled, and such non-random patterns have been shown to be stable through generations (see review by Stapley et al., 2017). In all plant species, there are regions of the genome that have few COs events; such regions are termed cold spots. The result of such linkage-disequilibrium is to maintain supergene groups, co-evolved loci or favorable linkage groups, which are of benefit to the plant. Alternatively, undesirable allele combinations could be maintained. As previously discussed, elevated temperature modulates the meiotic landscape of barley and *Arabidopsis* and disrupts linkage groups (Phillips et al., 2015; Lloyd et al., 2018; Modliszewski et al., 2018), which could alter the evolutionary fitness of the plants subjected to this stress.

Many of the anthropogenic stresses, including temperature, agrochemicals and fungicides were reported to cause spindle aberrations during the first and second meiotic division that in some instances gave rise to unreduced gametes and the potential for polyploidisation (Mason and Pires, 2015). High nutrient conditions aids the stabilization of neopolyploid and could drive the evolution of new polyploidy species. Xenobiotic compounds currently found in the environment have no equivalent in the history of our planet and therefore the long-term effect in terms of promoting polyploidy are unknown. The environmental climate of the planet has fluctuated on numerous occasions, and its impact on ploidy recognized. One such event occurred at the Cretaceous–Tertiary (KT) boundary that caused the mass extinction of many species, including about 60% of plant species, but drove the formation of polyploid species (Fawcett et al., 2009; Vanneste et al., 2014). Polyploidisation is believed to confer better adaptability and tolerance to altered environmental conditions, a trend that may be repeated in the current climate change cycle.

Plant speciation is often associated with structural changes in the genome resulting from aneuploidy, dysploidy, or other chromosome rearrangements such as translocations, inversions, fusions, or fissions (De Storme and Mason, 2014). None of the studies cited in this review report such structural changes, but none specifically set out to identify such morphological changes. Notable abnormal chromosome conformations, such as laggards, bridges, fragments, micronuclei, and aneuploidy are reported in many of the studies, and are capable of altering the genomic landscape of the subsequent generation.

## Summary

The influence of the human race on the natural environment is undeniable, ranging from altering climatic conditions globally to the local contamination with a specific xenobiotic compound. The influence of many such factors have been reported in a number of different angiosperms, with effects ranging from altered patterns of recombination to severe chromosome damage originating from stickiness and bridges. In most cases, only basic cytology has been used which has shed little light on the underlying mode of action of the factors. Many of the citations predate the development of the sensitive assays now available, and it would be profitable to use such methods to identify how these factors impinge upon on the biochemical pathways operating during meiosis, using concentrations of xenobiotics of relevance to plant populations. The long-term consequences of destabilizing the genome may have a profound evolutionary legacy.

## Author Contributions

All authors listed have made a substantial, direct and intellectual contribution to the work, and approved it for publication.

## Conflict of Interest Statement

The authors declare that the research was conducted in the absence of any commercial or financial relationships that could be construed as a potential conflict of interest.

## Footnotes

1 https://www.epa.gov/tsca-inventory

2 http://www.fao.org/faostat

## References

[B1] Adamakis I. D. Panteris E. Cherianidou A. Eleftheriou E. P. (2013). Effects of bisphenol A on the microtubule arrays in root meristematic cells of *Pisum sativum*. *Mutat. Res.* 750 111–120. 10.1016/j.mrgentox.2012.10.012 23174415

[B2] Aherne G. W. Hardcastle A. Nield A. H. (1990). Cytotoxic drugs and the aquatic environment: estimation of bleomycin in river and water samples. *J. Pharm. Pharmacol.* 42 741–742. 10.1111/j.2042-7158.1990.tb06574.x 1707456

[B3] Aldred K. J. Kerns R. J. Osheroff N. (2014). Mechanism of quinolone action and resistance. *Biochemistry* 53 1565–1574. 10.1021/bi5000564 24576155 PMC3985860

[B4] Allard P. Colaiacovo M. P. (2010). Bisphenol A impairs the double-strand break repair machinery in the germline and causes chromosome abnormalities. *Proc. Natl. Acad. Sci. U.S.A.* 2 20405–20410. 10.1073/pnas.1010386107 21059909 PMC2996676

[B5] Amer S. Ali E. (1983). Cytological effects of pesticides XIV. Effects of the insecticide Difterex “Trichlorophon” V. faba plant. *Cytologia* 48 761–770. 10.1508/cytologia.48.761

[B6] Amer S. M. Ali E. M. (1968). Cytological effects of pesticides: II. Meiotic effects of some phenols. *Cytologia* 33 21–33. 10.1508/cytologia.33.21 5655486

[B7] Amer S. M. Ali E. M. (1974). Cytological effects of pesticides V. Effects of some herbicides on *Vicia faba*. *Cytologia* 39 633–643. 10.1508/cytologia.39.633 4442286

[B8] Amer S. M. Farah O. R. (1968). Cytological effects of pesticides III. Meiotic effects of N-methyl-1-naphthyl carbamate “Sevin”. *Cytologia* 33 337–344. 10.1508/cytologia.33.337

[B9] Amer S. M. Farah O. R. (1976). Cytological effects of pesticides. VIII. Effects of the carbamate pesticides “IPC”, “Rogor”, and “Duphar” on *Vicia faba*. *Cytologia* 41 597–606. 10.1508/cytologia.41.597 1001037

[B10] Amer S. M. Farah O. R. (1980). Cytological effects of pesticides X. Meiotic effects of “Phosvel”. *Cytologia* 45 241–245. 10.1508/cytologia.45.241 26670031

[B11] Amer S. M. Farah O. R. (1983). Cytological effects of pesticides XIII. Meiotic effects of the insecticide “Dursban”. *Cytologia* 48 557–563. 10.1508/cytologia.48.557

[B12] Amer S. M. Farah O. R. (1987). Cytological effect of pesticides VIII Meiotic effects of the insecticide Methamidophos. *Cytologia* 52 303–307. 10.1508/cytologia.52.303

[B13] Andronic L. (2012). Viruses as triggers of DNA rearrangements in host plants. *Can. J. Plant Sci.* 92 1083–1091. 10.4141/cjps2011-197

[B14] Anis M. Wani A. A. (1997). Caffeine induced morpho-cytological variability in fenugreek, *Trigonella foenum-graecum.* *Cytologia* 62 343–349. 10.1508/cytologia.62.4_343

[B15] Aslam R. Bhat T. M. Choudhary S. Ansari M. Y. K. (2017). An overview on genotoxicity of heavy metal in a spice crop (*Capsicum annuum* L.) in respect to cyto-morphological behaviour. *Caryologia* 70 42–47. 10.1080/00087114.2016.1258884

[B16] Badr A. Hamoud M. Haroun S. (1987). Effect of the herbicide terbutryn on meiosis, yield and mitotic chromosomes in C 2 plants of *Vicia faba* L. *Biol. Plant.* 29 70–72. 10.1007/BF02902322

[B17] Bannon D. I. Drexler J. W. Fent G. M. Casteel S. W. Hunter P. J. Brattin W. J. (2009). Evaluation of small arms range soils for metal contamination and lead bioavailability. *Environ. Sci. Technol.* 43 9071–9076. 10.1021/es901834h 20000496

[B18] Barth S. Melchinger A. E. Devezi-Savula B. Lubberstedt T. (2000). A high-throughput system for genome-wide measurement of genetic recombination in *Arabidopsis thaliana* based on transgenic markers. *Funct. Integr. Genomics* 1 200–206. 10.1007/s101420000030 11793238

[B19] Bebber D. P. Holmes T. Gurr Sarah J. (2014). The global spread of crop pests and pathogens. *Global Ecol. Biogeogr.* 23 1398–1407. 10.1111/geb.12214

[B20] Bebber D. P. Ramotowski M. A. T. Gurr S. J. (2013). Crop pests and pathogens move polewards in a warming world. *Nat. Clim. Change* 3 985–988. 10.1038/nclimate1990

[B21] Bennett M. (1971). Effects of ethirimol on cytological characters in barley. *Nature* 230:406. 10.1038/230406a0 4927739

[B22] Bennett M. D. Rees H. (1970). Induced variation in chiasma frequency in rye in response to phosphate treatments. *Genet. Res.* 16 325–331. 10.1017/S0016672300002585

[B23] Bert V. Bonnin I. Saumitou-Laprade P. De Laguérie P. Petit D. (2002). Do *Arabidopsis halleri* from nonmetallicolous populations accumulate zinc and cadmium more effectively than those from metallicolous populations? *New Phytol.* 155 47–57. 10.1046/j.1469-8137.2002.00432.x 33873296

[B24] Bomblies K. Higgins J. D. Yant L. (2015). Meiosis evolves: adaptation to external and internal environments. *New Phytol.* 208 306–323. 10.1111/nph.13499 26075313

[B25] Boyko A. Hudson D. Bhomkar P. Kathiria P. Kovalchuk I. (2006). Increase of homologous recombination frequency in vascular tissue of Arabidopsis plants exposed to salt stress. *Plant Cell. Physiol.* 47 736–742. 10.1093/pcp/pcj045 16608867

[B26] Bruton T. Alboloushi A. de la Garza B. Kim B.-O. Halden R. U. (2010). “Fate of caffeine in the environment and ecotoxicological considerations,” in *Proceedings of the Contaminants of Emerging Concern in the Environment: Ecological and Human Health Considerations* (Washington, DC: American Chemical Society) 257.

[B27] Bykova O. Limousin J. M. Ourcival J. M. Chuine I. (2018). Water deficit disrupts male gametophyte development in *Quercus ilex*. *Plant Biol.* 20 450–455. 10.1111/plb.12692 29350475

[B28] Caldwell J. (1952). Some effects of a plant virus on nuclear division. *Ann. Appl. Biol.* 39 98–102. 10.1111/j.1744-7348.1952.tb01001.x

[B29] Cardinale B. Duffy J. Gonzalez A. Hooper D. Perrings C. Venail P. (2012). Biodiversity loss and its impact on humanity. *Nature* 486 59–67. 10.1038/nature11148 22678280

[B30] Casteel C. L. Segal L. M. Niziolek O. K. Berenbaum M. R. DeLucia E. H. (2012). Elevated carbon dioxide increases salicylic acid in *Glycine max*. *Environ. Entomol.* 41 1435–1442. 10.1603/EN12196 23321090

[B31] Choudhary S. Sajid S. (1986). The behaviour of meiotic chromosomes as revealed through the use of Bavistin on pea. *Cytologia* 51 279–288. 10.1508/cytologia.51.279

[B32] Currie R. S. Liang G. H. (1996). Cytological and morphological effects of atrazine and propazine application on grain sorghum. *Cytologia* 61 359–363. 10.1508/cytologia.61.359

[B33] Davis A. P. Shokouhian M. Ni S. (2001). Loading estimates of lead, copper, cadmium, and zinc in urban runoff from specific sources. *Chemosphere* 44 997–1009. 10.1016/S0045-6535(00)00561-0 11513434

[B34] de la Peña A. Puertas M. J. Merino F. (1981). Bimeiosis induced by caffeine. *Chromosoma* 83 241–248. 10.1007/BF00286792

[B35] De Storme N. Geelen D. (2014). The impact of environmental stress on male reproductive development in plants: biological processes and molecular mechanisms. *Plant Cell Environ.* 37 1–18. 10.1111/pce.12142 23731015 PMC4280902

[B36] De Storme N. Mason A. (2014). Plant speciation through chromosome instability and ploidy change: cellular mechanisms, molecular factors and evolutionary relevance. *Curr. Plant Biol.* 1 10–33. 10.1016/j.cpb.2014.09.002

[B37] Deniz B. Tufan A. (1998). The influence of phosphate treatment on chromosome segregations and tetrad regularity and using tetrads in determination of regular meiosis diploid. *Turk. J. Agric. For.* 22 381–390.

[B38] Devadas N. Rajam M. V. Subhash K. (1986). Comparative mutagenicity of four organophosphorus insecticides in meiotic system of red pepper. *Cytologia* 51 645–653. 10.1508/cytologia.51.645

[B39] Dhesi J. (1975). Effect of minerals on the chiasma frequency in desynaptic pearl millet. *Curr. Sci.* 44 862–863.

[B40] Donner E. Eriksson E. Holten-Luetzhoft H.-C. Scholes L. Revitt M. Ledin A. (2010). “Identifying and classifying the sources and uses of xenobiotics in urban environments,” in *Xenobiotics in the Urban Water Cycle: Mass Flows, Environmental Processes, Mitigation and Treatment Strategies* eds Fatta-Kassinos D. Bester K. Kümmerer K. (Dordrecht: Springer) 27–50. 10.1007/978-90-481-3509-7_2

[B41] Dowrick G. J. (1957). The influence of temperature on meiosis. *Heredity* 11 37–49. 10.1038/hdy.1957.4

[B42] Elliott C. G. (1955). The effect of temperature on chiasma frequency. *Heredity* 9 385–398. 10.1038/hdy.1955.39

[B43] Evans N. Baierl A. Semenov M. A. Gladders P. Fitt B. D. L. (2008). Range and severity of a plant disease increased by global warming. *J. R. Soc. Interface* 5 525–531. 10.1098/rsif.2007.1136 17711818 PMC3226977

[B44] Evans-Roberts K. M. Mitchenall L. A. Wall M. K. Leroux J. Mylne J. S. Maxwell A. (2016). DNA Gyrase is the target for the quinolone drug ciprofloxacin in *Arabidopsis thaliana*. *J. Biol. Chem.* 291 3136–3144. 10.1074/jbc.M115.689554 26663076 PMC4751362

[B45] Fawcett J. A. Maere S. Van de Peer Y. (2009). Plants with double genomes might have had a better chance to survive the Cretaceous-Tertiary extinction event. *Proc. Natl. Acad. Sci. U.S.A.* 106 5737–5742. 10.1073/pnas.0900906106 19325131 PMC2667025

[B46] Fedak G. (1973). Increased chiasma frequency in desynaptic barley in response to phosphate treatments. *Can. J. Genet. Cytol.* 15 647–649. 10.1139/g73-076 4762792

[B47] Fisun K. Rasgele P. G. (2009). Genotoxic effects of raxil on root tips and anthers of *Allium cepa* L. *Caryologia* 62 1–9. 10.1080/00087114.2004.10589659

[B48] Food and Agriculture Organization [FAO] (2017). *World Fertilizer Trends and Outlook to 2020.* Available at: http://www.fao.org/publications

[B49] Francis K. E. Lam S. Y. Harrison B. Bey A. L. Berchowitz L. E. Copenhaver G. P. (2007). Pollen tetrad-based visual assay for meiotic recombination in *Arabidopsis*. *Proc. Natl. Acad. Sci. U.S.A.* 104 3913–3918. 10.1073/pnas.0608936104 17360452 PMC1805420

[B50] Gaffney J. S. Marley N. A. (2009). The impacts of combustion emissions on air quality and climate – From coal to biofuels and beyond. *Atmos. Environ.* 43 23–36. 10.1016/j.atmosenv.2008.09.016

[B51] Gale M. D. Rees H. (1970). Genes controlling chiasma frequency in *Hordeum*. *Heredity* 25 393–410. 10.1038/hdy.1970.40

[B52] Gaw S. Thomas K. V. Hutchinson T. H. (2014). Sources, impacts and trends of pharmaceuticals in the marine and coastal environment. *Philos. Trans. R. Soc. B* 369:20130572. 10.1098/rstb.2013.0572 25405962 PMC4213585

[B53] Gely-Pernot A. Hao C. Becker E. Stuparevic I. Kervarrec C. Chalmel F. (2015). The epigenetic processes of meiosis in male mice are broadly affected by the widely used herbicide atrazine. *BMC Genomics* 16:885. 10.1186/s12864-015-2095-y 26518232 PMC4628360

[B54] Gely-Pernot A. Saci S. Kernanec P.-Y. Hao C. Giton F. Kervarrec C. (2017). Embryonic exposure to the widely-used herbicide atrazine disrupts meiosis and normal follicle formation in female mice. *Sci. Rep.* 7:3526. 10.1038/s41598-017-03738-1 28615648 PMC5471253

[B55] George N. M. (2000). Evaluation on mutagenic effects of the three heavy metals on *Vicia faba* plants. *Cytologia* 65 75–82. 10.1508/cytologia.65.75 16376989

[B56] Golet E. M. Strehler A. Alder A. C. Giger W. (2002). Determination of fluoroquinolone antibacterial agents in sewage sludge and sludge-treated soil using accelerated solvent extraction followed by solid-phase extraction. *Anal. Chem.* 74 5455–5462. 10.1021/ac025762m 12433073

[B57] Goulas A. Haudin C. S. Bergheaud V. Dumeny V. Ferhi S. Nelieu S. (2016). A new extraction method to assess the environmental availability of ciprofloxacin in agricultural soils amended with exogenous organic matter. *Chemosphere* 165 460–469. 10.1016/j.chemosphere.2016.09.040 27677122

[B58] Grant V. (1953). Cytogenetics of the hybrid Gilia millefoliata × achilleaefolia. *Chromosoma* 5 372–390. 10.1007/BF01271494 13067195

[B59] Griffing B. Langridge J. (1963). Factors affecting crossing over in the tomato. *Aust. J. Biol. Sci.* 16 826–837. 10.1071/BI9630826 18086712

[B60] Hamscher G. Sczesny S. Höper H. Nau H. (2002). Determination of persistent tetracycline residues in soil fertilized with liquid manure by high-performance liquid chromatography with electrospray ionization tandem mass spectrometry. *Anal. Chem.* 74 1509–1518. 10.1021/ac015588m 12033238

[B61] Hanson W. (1961). Effect of calcium and phosphorus nutrition on genetic recombination in the soybean. *Crop Sci.* 1 384–384. 10.2135/cropsci1961.0011183X000100050033x

[B62] Hautier Y. Tilman D. Isbell F. Seabloom E. Borer E. Reich P. (2015). Anthropogenic environmental changes affect ecosystem stability via biodiversity. *Science* 348 336–340. 10.1126/science.aaa1788 25883357

[B63] Heberer T. (2002). Tracking persistent pharmaceutical residues from municipal sewage to drinking water. *J. Hydrol.* 266 175–189. 10.1016/S0022-1694(02)00165-8 12227607

[B64] Higgins J. D. Perry R. M. Barakate A. Ramsay L. Waugh R. Halpin C. (2012). Spatiotemporal asymmetry of the meiotic program underlies the predominantly distal distribution of meiotic crossovers in barley. *Plant Cell* 24 4096–4109. 10.1105/tpc.112.102483 23104831 PMC3517238

[B65] Hossain M. G. (1978). Effects of external environmental factors on chromosome pairing in autotetraploid rye. *Cytologia* 43 21–34. 10.1508/cytologia.43.21

[B66] Hulskotte J. H. J. Denier van der Gon H. A. C. Visschedijk A. J. H. Schaap M. (2007). Brake wear from vehicles as an important source of diffuse copper pollution. *Water Sci. Technol.* 56 223–231. 10.2166/wst.2007.456 17711019

[B67] International Plant Protection Convention [IPPC] (2014). *Climate Change 2014: Synthesis Report. Contribution of Working Groups I, II and III to the Fifth Assessment Report of the Intergovernmental Panel on Climate Change.* Geneva: IPCC.

[B68] Jensen J. (1981). Effects of temperature on genetic recombination in barley. *Hereditas* 94 215–218. 10.1111/j.1601-5223.1981.tb01755.x

[B69] Jones R. A. (2016). Future scenarios for plant virus pathogens as climate change progresses. *Adv. Virus Res.* 95 87–147. 10.1016/bs.aivir.2016.02.004 27112281

[B70] Karihaloo J. L. (1986). Stress induced desynapsis in *Hemerocallis*. *Cytologia* 51 193–199. 10.1508/cytologia.51.193

[B71] Karihaloo J. L. (1994). Genotype-environment interaction in the desynapsis of a *Hemerocallis* ‘Sleeping Beauty’ clone and its progeny. *Cytologia* 59 483–491. 10.1508/cytologia.59.483 24420126

[B72] Kathiria P. Sidler C. Golubov A. Kalischuk M. Kawchuk L. M. Kovalchuk I. (2010). Tobacco Mosaic Virus infection results in an increase in recombination frequency and resistance to viral, bacterial, and fungal pathogens in the progeny of infected tobacco plants. *Plant Physiol.* 153 1859–1870. 10.1104/pp.110.157263 20498336 PMC2923882

[B73] Kaul B. L. (1968). A study of meiosis in virus infected *Datura quarcifolia*. *Cytologia* 33 17–20. 10.1508/cytologia.33.17

[B74] Kaymak F. (2005). Cytogenetic effects of maleic hydrazide on *Helianthus annuus* L. *Pak. J. Biol. Sci.* 8 104–108. 10.3923/pjbs.2005.104.108

[B75] Khan S. Cao Q. Zheng Y. M. Huang Y. Z. Zhu Y. G. (2008). Health risks of heavy metals in contaminated soils and food crops irrigated with wastewater in Beijing, China. *Environ. Pollut.* 152 686–692. 10.1016/j.envpol.2007.06.056 17720286

[B76] Koti S. Reddy K. R. Reddy V. R. Kakani V. G. Zhao D. (2005). Interactive effects of carbon dioxide, temperature, and ultraviolet-B radiation on soybean (*Glycine max* L.) flower and pollen morphology, pollen production, germination, and tube lengths. *J. Exp. Bot.* 56 725–736. 10.1093/jxb/eri044 15611147

[B77] Kovalchuk I. Kovalchuk O. Kalck V. Boyko V. Filkowski J. Heinlein M. (2003). Pathogen-induced systemic plant signal triggers DNA rearrangements. *Nature* 423 760–762. 10.1038/nature01683 12802336

[B78] Kristen U. (1997). Use of higher plants as screens for toxicity assessment. *Toxicol. In Vitro* 11 181–191. 10.1016/S0887-2333(97)00005-2 20654304

[B79] Kuchy A. Wani A. Kamili A. (2016). Cytogenetic effects of three commercially formulated pesticides on somatic and germ cells of *Allium cepa*. *Environ. Sci. Pollut. R.* 23 6895–6906. 10.1007/s11356-015-5912-6 26670031

[B80] Kumar G. (2007). Comparative genotoxic potential of mercury and cadmium in soybean. *Turk. J. Biol.* 31 13–18.

[B81] Kumar G. Rai P. K. (2010). The genotoxic potential of two heavy metals in inbred lines of maize (*Zea mays*). *Turk. J. Bot.* 34 39–46.

[B82] Kumar G. Ritambhara T. (2006). Individual and combined effects of lead and gamma irradiations on genetic recombination in *Lathyrus sativus*. *J. Phytol. Res.* 19 215–220.

[B83] Kumar U. Sinha S. S. N. (1991). Genotoxic effects of two pesticides (Rogor and Bavistin) and an antibiotic (Streptomycin) in meiotic cells of grasspea (*Lathyrus sativus* L.). *Cytologia* 56 209–214. 10.1508/cytologia.56.209

[B84] Kumar Ravindra P. (2017). Studies on pollen viability of papaya (Carica papaya) infected by Papaya Ring Spot Virus (PRSV). *Int. J. Adv. Res.* 5 1664–1667. 10.21474/IJAR01/4585

[B85] Küster A. Adler N. (2014). Pharmaceuticals in the environment: scientific evidence of risks and its regulation. *Philos. Trans. R. Soc. B* 369:20130587. 10.1098/rstb.2013.0587 25405974 PMC4213597

[B86] Lakshmi N. Prakash N. Harini I. (1988). Cytological effects of agricultural chemicals I. effects of insecticides “Ekalux and Metasystox” on chilli (*Capsicum annuum* L.). *Cytologia* 53 703–708. 10.1508/cytologia.53.703

[B87] Law C. (1963). An effect of potassium on chiasma frequency and recombination. *Genetica* 33 313–329. 10.1007/BF01725768

[B88] Lee H. B. Peart T. E. Svoboda M. L. (2007). Determination of ofloxacin, norfloxacin, and ciprofloxacin in sewage by selective solid-phase extraction, liquid chromatography with fluorescence detection, and liquid chromatography-tandem mass spectrometry. *J. Chromatogr. A* 1139 45–52. 10.1016/j.chroma.2006.11.068 17157863

[B89] Lee K. Rao G. Barnett F. Liang G. (1974). Further evidence of meiotic instability induced by Atrazine in grain-sorghum. *Cytologia* 39 697–702. 10.1508/cytologia.39.697

[B90] Liang G. H. Feltner K. Russ O. (1969). Meiotic and morphological response of grain sorghum to atrazine, 2, 4-D, oil, and their combinations. *Weed Sci.* 17 8–12.

[B91] Lin Y. J. (1982). Temperature and chiasma formation in *Rhoeo spathacea* var. variegata. *Genetica* 60 25–30. 10.1007/BF00121453

[B92] Lloyd A. Morgan C. Franklin C. Bomblies K. (2018). Plasticity of meiotic recombination rates in response to temperature in *Arabidopsis*. *Genetics* 208 1409–1420. 10.1534/genetics.117.300588 29496746 PMC5887139

[B93] Lucht J. M. Mauch-Mani B. Steiner H. Y. Metraux J. P. Ryals J. Hohn B. (2002). Pathogen stress increases somatic recombination frequency in Arabidopsis. *Nat. Genet.* 30 311–314. 10.1038/ng846 11836502

[B94] Ma T.-H. Anderson V. A. Ahmed I. (1982). “Environmental clastogens detected by meiotic pollen mother cells of Tradescantia,” in *Genotoxic Effects of Airborne Agents* eds Tice R. Raymond Daniel Schaich Karen M. (Boston, MA: Springer) 141–157.

[B95] Ma T.-H. Harris M. (1987a). Tradescantia micronucleus (Trad-MCN) assay - a potential indoor pollution monitor. *Environ. Mutagen.* 9(Suppl.):8.

[B96] Ma T.-H. Harris M. (1987b). “Tradescantia micronucleus (Trad-MCN) bioassay- a promising indoor air pollution monitoring system,” in *Proceedings of the 4th International Conference on Indoor Air Quality and Climate* Berlin 243–247.

[B97] Ma T.-H. Van Anderson A. Sandhu S. S. (1980). “A preliminary study of the clastogenic effects of diesel exhaust fumes using the tradescantia micronucleus bioassay,” in *Short-Term Bioassays in the Analysis of Complex Environmental Mixtures II* eds Waters M. Nesnow S. Huisingh J. L. Sandhu S. S. Claxton L. (Boston, MA: Springer) 351–358.

[B98] Mann S. K. (1977). Cytological and genetic effects of dithane fungicides on *Allium cepa*. *Environ. Exp. Bot.* 1977 7–12. 10.1016/0098-8472(77)90014-4

[B99] Mann S. K. (1978). Interaction of tetracycline (TC) with chromosomes in *Allium cepa*. *Environ. Exp. Bot.* 18 201–205. 10.1016/0098-8472(78)90039-4

[B100] Martín A. C. Rey M.-D. Shaw P. Moore G. (2017). Dual effect of the wheat Ph1 locus on chromosome synapsis and crossover. *Chromosoma* 126 669–680. 10.1007/s00412-017-0630-0 28365783 PMC5688220

[B101] Mason A. S. Pires J. C. (2015). Unreduced gametes: meiotic mishap or evolutionary mechanism? *Trends Genet.* 31 5–10. 10.1016/j.tig.2014.09.011 25445549

[B102] Mickelbart M. V. Hasegawa P. M. Bailey-Serres J. (2015). Genetic mechanisms of abiotic stress tolerance that translate to crop yield stability. *Nat. Rev. Genet.* 16 237–251. 10.1038/nrg3901 25752530

[B103] Mittal N. Srivastava A. K. (2014). Cadmium and chromium induced aberrations in the reproductive biology of *Hordeum vulgare*. *Cytologia* 79 207–214. 10.1508/cytologia.79.207

[B104] Modliszewski J. L. Copenhaver G. P. (2017). Meiotic recombination gets stressed out: CO frequency is plastic under pressure. *Curr. Opin. Plant Biol.* 36 95–102. 10.1016/j.pbi.2016.11.019 28258986

[B105] Modliszewski J. L. Wang H. Albright A. R. Lewis S. M. Bennett A. R. Huang J. (2018). Elevated temperature increases meiotic crossover frequency via the interfering (Type I) pathway in *Arabidopsis thaliana*. *PLoS Genet.* 14:e1007384. 10.1371/journal.pgen.1007384 29771908 PMC5976207

[B106] Molinier J. Ries G. Zipfel C. Hohn B. (2006). Transgeneration memory of stress in plants. *Nature* 442 1046–1049. 10.1038/nature05022 16892047

[B107] Moss B. (2008). Water pollution by agriculture. *Philos. Trans. R. Soc. Lond. B Biol. Sci.* 363 659–666. 10.1098/rstb.2007.2176 17666391 PMC2610176

[B108] Namuco O. S. O’Toole J. C. (1986). Reproductive stage water stress and sterility. I. Effect of stress during meiosis I. *Crop Sci.* 26 317–321. 10.2135/cropsci1986.0011183X002600020022x

[B109] Naveedullah Hashmi M. Z. Yu C. Shen H. Duan D. Shen C. (2013). Risk assessment of heavy metals pollution in agricultural soils of siling reservoir watershed in Zhejiang province, China. *Biomed. Res. Int.* 2013:590306. 10.1155/2013/590306 24151611 PMC3787591

[B110] Noedler K. Licha T. Voutsa D. (2013). Twenty years later - Atrazine concentrations in selected coastal waters of the Mediterranean and the Baltic Sea. *Mar. Pollut. Bull.* 70 112–118. 10.1016/j.marpolbul.2013.02.018 23481690

[B111] Omidi M. Siahpoosh M. R. Mamghani R. Modarresi M. (2014). The influence of terminal heat stress on meiosis abnormalities in pollen mother cells of wheat. *Cytologia* 79 49–58. 10.1508/cytologia.79.49

[B112] Pandey R. (2017). Studies on pollen viability of papaya (Carica papaya) infected by papaya ring spot virus (prsv). *Int. J. Adv. Res.* 5 1664–1667. 10.21474/IJAR01/4585

[B113] Pecrix Y. Rallo G. Folzer H. Cigna M. Gudin S. Le Bris M. (2011). Polyploidization mechanisms: temperature environment can induce diploid gamete formation in *Rosa* sp. *J. Exp. Bot.* 62 3587–3597. 10.1093/jxb/err052 21398431

[B114] Phillips D. Jenkins G. Macaulay M. Nibau C. Wnetrzak J. Fallding D. (2015). The effect of temperature on the male and female recombination landscape of barley. *New Phytol.* 208 421–429. 10.1111/nph.13548 26255865

[B115] Powell J. B. Nilan R. A. (1963). Influence of temperature on crossing over in an inversion heterozygote in barley. *Crop Sci.* 3 11–13. 10.2135/cropsci1963.0011183X000300010005x

[B116] Prakash N. Lakshmi N. Harini I. (1988). Cytological effects of agricultural chemicals. II. Effects of fungicides” Bavistin” and” Deltan” on chilli (*Capsicum annuum* L.). *Cytologia* 53 709–715. 10.1508/cytologia.53.709

[B117] Prakken R. (1943). Studies of asynapsis in rye. *Hereditas* 29 475–495. 10.1111/j.1601-5223.1943.tb03301.x 17249107

[B118] Qi W. Müller B. Pernet-Coudrier B. Singer H. Liu H. Qu J. (2014). Organic micropollutants in the Yangtze River: seasonal occurrence and annual loads. *Sci. Total Environ.* 472 789–799. 10.1016/j.scitotenv.2013.11.019 24334001

[B119] Rahavi M. R. Migicovsky Z. Titov V. Kovalchuk I. (2011). Transgenerational adaptation to heavy metal salts in *Arabidopsis*. *Front. Plant Sci.* 2:91. 10.3389/fpls.2011.00091 22639617 PMC3355606

[B120] Razu M. Zaman S. Akhter R. Rahman M. Rahaman M. H. Mazid M. (2014). Morphological and cytological effects of two herbicides on tetraploid wheat (*Triticum durum* L.). *J. Biosci.* 20 143–151.

[B121] Reddy S. S. Rao G. M. (1981). Cytogenetic effects of agricultural chemicals I. Effects of insecticides” BHC and nuvacron” on chromosomal mechanism in relation to yield and yield components in chilli (*Capsicum annuum* L.). *Cytologia* 46 699–707. 10.1508/cytologia.46.699

[B122] Rey M. D. Martin A. C. Smedley M. Hayta S. Harwood W. Shaw P. (2018). Magnesium increases homoeologous crossover frequency during meiosis in ZIP4 (Ph1 gene) mutant wheat-wild relative hybrids. *Front. Plant Sci.* 9:509. 10.3389/fpls.2018.00509 29731763 PMC5920029

[B123] Rezaei M. Arzani A. Sayed-Tabatabaei B. E. (2010). Meiotic behaviour of tetraploid wheats (*Triticum turgidum* L.) and their synthetic hexaploid wheat derivates influencedby meiotic restitution and heat stress. *J. Genet.* 89 401–407. 10.1007/s12041-010-0058-2 21273690

[B124] Robinson B. H. Bischofberger S. Stoll A. Schroer D. Furrer G. Roulier S. (2008). Plant uptake of trace elements on a Swiss military shooting range: uptake pathways and land management implications. *Environ. Pollut.* 153 668–676. 10.1016/j.envpol.2007.08.034 17949872

[B125] Rodrigues G. S. Ma T. H. Pimentel D. Weinstein L. H. (1997). Tradescantia bioassays as monitoring systems for environmental mutagenesis: a review. *Crit. Rev. Plant Sci.* 16 325–359. 10.1080/07352689709701953 367768

[B126] Rodrigues G. S. Madkour S. A. Weinstein L. H. (1996). Genotoxic activity of ozone in *Tradescantia*. *Environ. Exp. Bot.* 36 45–50. 10.1016/0098-8472(95)00042-9

[B127] Rowan B. A. Oldenburg D. J. Bendich A. J. (2010). RecA maintains the integrity of chloroplast DNA molecules in *Arabidopsis*. *J. Exp. Bot.* 61 2575–2588. 10.1093/jxb/erq088 20406785 PMC2882256

[B128] Saini H. S. Sedgley M. Aspinall D. (1984). Developmental anatomy in wheat of male sterility induced by heat stress, water deficit or abscisic acid. *Aust. J. Plant Physiol.* 11 243–253. 10.1071/PP9840243

[B129] Shan Y. Tysklind M. Hao F. Ouyang W. Chen S. Lin C. (2013). Identification of sources of heavy metals in agricultural soils using multivariate analysis and GIS. *J. Soils Sedim.* 13 720–729. 10.1007/s11368-012-0637-3

[B130] Sharma C. Behera B. N. Raju D. S. S. Rao B. G. S. (1983). Effects of some systemic fungicides on the chiasma frequencies in *Hordeum vulgare*. *Cytologia* 48 749–752. 10.1508/cytologia.48.749

[B131] Sharma C. Patra B. Raju D. S. S. Murty K. V. (1981). Chiasma variation in *Hordeum vulgare* after exposure to herbicides. *Mutat. Res.* 91 333–336. 10.1016/0165-7992(81)90010-5

[B132] Sharma C. B. S. Panneerselvam N. (1990). Genetic toxicology of pesticides in higher-plant systems. *Crit. Rev. Plant Sci.* 9 409–442. 10.1016/j.mrfmmm.2016.08.003 27566293

[B133] Si W. Yuan Y. Huang J. Zhang X. Zhang Y. Zhang Y. (2015). Widely distributed hot and cold spots in meiotic recombination as shown by the sequencing of rice F2 plants. *New Phytol.* 206 1491–1502. 10.1111/nph.13319 25664766

[B134] Singh N. Srivastava A. (2014). Biomonitoring of genotoxic effect of glyphosate and pendimethalin in *Vigna mungo* populations. *Cytologia* 79 173–180. 10.1508/cytologia.79.173

[B135] Skazkin F. D. Zavadskaya I. G. (1957). On the influence of soil moisture deficiency and nitrogen nutrition on microsporogenesis in barley. *Dokl Akad Nauk SSSR* 117 240–242.

[B136] Soriano J. D. (1984). Herbicide-induced chromosomal aberrations and inheritance of a digenic seedling mutation in sorghum. *Cytologia* 49 201–207. 10.1508/cytologia.49.201

[B137] Srivastava N. Kumar G. (2016). Effect of waterlogging stress on meiotic course, tetrad formation and pollen fertility of Sesbania pea. *Cytol. Genet.* 50 36–39. 10.3103/S0095452716010096 27266183

[B138] Stapley J. Feulner P. Johnston S. Santure A. Smadja C. (2017). Variation in recombination frequency and distribution across eukaryotes: patterns and processes. *Philos. T. R. Soc. B.* 372:20160455. 10.1098/rstb.2016.0455 29109219 PMC5698618

[B139] Swaminathan M. S. Ninan T. Magoon M. L. (1959). Cytological effects of mosaic virus in chillis. *Genetica* 30 63–69. 10.1007/BF01535665 13836008

[B140] Thiele-Bruhn S. (2003). Pharmaceutical antibiotic compounds in soils – a review. *J. Plant Nutr. Soil Sci.* 166 145–167. 10.1002/jpln.200390023

[B141] Tian M. Liu Y. Zhang Y. Kang X. Zhang P. (2018). High temperature exposure did not affect induced 2n pollen viability in *Populus*. *Plant Cell Environ.* 41 1383–1393. 10.1111/pce.13165 29430685

[B142] Tilman D. Cassman K. G. Matson P. A. Naylor R. Polasky S. (2002). Agricultural sustainability and intensive production practices. *Nature* 418 671–677. 10.1038/nature01014 12167873

[B143] Tornero V. Hanke G. (2016). Chemical contaminants entering the marine environment from sea-based sources: a review with a focus on European seas. *Mar. Pollut. Bull.* 112 17–38. 10.1016/j.marpolbul.2016.06.091 27444857

[B144] Trebicki P. Dader B. Vassiliadis S. Fereres A. (2017). Insect-plant-pathogen interactions as shaped by future climate: effects on biology, distribution, and implications for agriculture. *Insect Sci.* 24 975–989. 10.1111/1744-7917.12531 28843026

[B145] Tripathi R. Girjesh K. (2010). Genetic loss through heavy metal induced chromosomal stickiness in grass pea. *Caryologia* 63 223–228. 10.1080/00087114.2010.10589731

[B146] Uslu M. O. Jasim S. Arvai A. Bewtra J. Biswas N. (2013). A survey of occurrence and risk assessment of pharmaceutical substances in the Great Lakes basin. *Ozone Sci. Eng.* 35 249–262. 10.1080/01919512.2013.793595

[B147] van Tol N. Rolloos M. van Loon P. van der Zaal B. J. (2018). MeioSeed: a CellProfiler-based program to count fluorescent seeds for crossover frequency analysis in *Arabidopsis thaliana*. *Plant Methods* 18 14–32. 10.1186/s13007-018-0298-3 29692862 PMC5905130

[B148] Vanneste K. Baele G. Maere S. Van de Peer Y. (2014). Analysis of 41 plant genomes supports a wave of successful genome duplications in association with the Cretaceous-Paleogene boundary. *Genome Res.* 24 1334–1347. 10.1101/gr.168997.113 24835588 PMC4120086

[B149] Verde L. A. (2003). *The Effect of Stress on Meiotic Recombination in Maize (Zea mays L).* Ph.D. thesis, Iowa State University, Ames, IA.

[B150] Vonberg D. Hofmann D. Vanderborght J. Lelickens A. Koeppchen S. Puetz T. (2014). Atrazine soil core residue analysis from an agricultural field 21 years after its ban. *J. Environ. Qual.* 43 1450–1459. 10.2134/jeq2013.12.0497 25603092

[B151] Wang J. Daili Li D. Shang F. Kang X. (2017). High temperature-induced production of unreduced pollen and its cytological effects in *Populus*. *Nat. Sci. Rep.* 7:5281. 10.1038/s41598-017-05661-x 28706219 PMC5509662

[B152] Wang X. Copenhaver G. P. (2018). Meiotic recombination: mixing it up in plants. *Annu. Rev. Plant Biol.* 69 13.11–13.33. 10.1146/annurev-arplant-042817-040431 29489392

[B153] Williams C. F. McLain J. E. (2012). Soil persistence and fate of carbamazepine, lincomycin, caffeine, and ibuprofen from wastewater reuse. *J. Environ. Qual.* 41 1473–1480. 10.2134/jeq2011.0353 23099938

[B154] Wilson J. Y. (1959a). Chiasma frequency in relation to temperature. *Genetica* 29 290–303. 10.1007/BF01535715

[B155] Wilson J. Y. (1959b). Temperature effect on chiasma frequency in the bluebell *Endymion nonscriptus*. *Chromosoma* 10 337–354. 10.1007/BF00396577

[B156] Wuu K. (1967). Chromosomal aberrations induced by pesticides in meiotic cells of barley. *Cytologia* 32 31–41. 10.1508/cytologia.32.31

[B157] Wuu K. Grant W. (1966). Induced abnormal meiotic behavior in a barley plant (*Hordeum vulgare* L) with herbicide Lorox. *Phyton* 23:63.

[B158] Xu X. Zhao Y. Zhao X. Wang Y. Deng W. (2014). Sources of heavy metal pollution in agricultural soils of a rapidly industrializing area in the Yangtze Delta of China. *Ecotoxicol. Environ. Saf.* 108 161–167. 10.1016/j.ecoenv.2014.07.001 25063882

[B159] Yao Y. Kathiria P. Kovalchuk I. (2013). A systemic increase in the recombination frequency upon local infection of *Arabidopsis thaliana* plants with oilseed rape mosaic virus depends on plant age, the initial inoculum concentration and the time for virus replication. *Front. Plant Sci.* 4:61. 10.3389/fpls.2013.00061 23519399 PMC3604634

[B160] Yelina N. Diaz P. Lambing C. Henderson I. R. (2015). Epigenetic control of meiotic recombination in plants. *Sci. China Life Sci.* 58 223–231. 10.1007/s11427-015-4811-x 25651968

[B161] Zhang H. Zhou Y. Huang Y. Wu L. Liu X. Luo Y. (2016). Residues and risks of veterinary antibiotics in protected vegetable soils following application of different manures. *Chemosphere* 152 229–237. 10.1016/j.chemosphere.2016.02.111 26971176

[B162] Zhang M.-K. Liu Z.-Y. Wang H. (2010). Use of single extraction methods to predict bioavailability of heavy metals in polluted soils to rice. *Commun. Soil Sci. Plant* 41 820–831. 10.1080/00103621003592341

[B163] Zhang S. Li X. Sun Z. Shao S. Hu L. Ye M. (2015). Antagonism between phytohormone signalling underlies the variation in disease susceptibility of tomato plants under elevated CO2. *J. Exp. Bot.* 66 1951–1963. 10.1093/jxb/eru538 25657213 PMC4378629

[B164] Zheng T. Nibau C. Phillips D. W. Jenkins G. Armstrong S. J. Doonan J. H. (2014). CDKG1 protein kinase is essential for synapsis and male meiosis at high ambient temperature in *Arabidopsis thaliana*. *Proc. Natl. Acad. Sci. U.S.A.* 111 2182–2187. 10.1073/pnas.1318460111 24469829 PMC3926089

[B165] Ziolkowski P. A. Underwood C. J. Lambing C. Martinez-Garcia M. Lawrence E. J. Ziolkowska L. (2017). Natural variation and dosage of the HEI10 meiotic E3 ligase control *Arabidopsis* crossover recombination. *Genes Dev.* 31 306–317. 10.1101/gad.295501.116 28223312 PMC5358726

[B166] Zohair S. Khatoon S. Zaidi S. (2012). Cytological studies on 14 plant species under polluted conditions. *Pak. J. Bot.* 44 1977–1982.

